# “Now once again this idea of yours (…) how does it sound when I say that?” – Changing the perspective: how coach’s questioning practices elicit self-reflecting processes in clients

**DOI:** 10.3389/fpsyg.2024.1241489

**Published:** 2024-03-14

**Authors:** Chantal Moos, Thomas Spranz-Fogasy

**Affiliations:** Leibniz Institute for the German Language, Mannheim, Germany

**Keywords:** business coaching, questioning sequences, Transformative Sequences, self-reflection, formulations, perspective

## Abstract

Drawing upon the transformative power of questions, the paper investigates questioning sequences from authentic coaching data to examine the systematic use of a particular succession of formulation and question and its impact on inviting self-reflection processes in the client and eliciting change. The object of investigation in this paper are therefore questioning sequences in which a coach asks a question immediately after a rephrasing or relocating action, prompting the client to respond in an explicit or implicit way. The coach hereby shifts the focus to a hypothetical scenario, prompting the client to change her perspective on the matter and reflect on her own statements, ideas and attitudes from an outside perspective. The paper aims to contribute to closing the research gap of the change potential of reflection-stimulating action techniques used by coaches, by investigating one of many ways of how questions can be powerful tools to invite a change of perspective for the client. The study focuses on one coaching process consisting of three sessions between a female coach and a female client, utilizing a single case study approach. The data collection was part of the interdisciplinary project “Questioning Sequences in Coaching”, comprising 14 authentic coaching processes. The analysis follows Peräkylä’s Transformative Sequences model, examining the first position including the formulation and the subsequent question, the client’s response, and the coach’s reaction to the response. On a practical level, the main purpose of this paper is not to contribute to the many ways practical literature recommends coaches how to do their work and how to ask questions, but rather to show in what ways the elicitation of self-reflection processes in clients has been achieved by other coaches in authentic coaching sessions.

## 1 Introduction

Coaching is a dynamic, transformative practice aimed at cultivating self-reflection with the ultimate objective of eliciting change. Much like in other helping professions, coaching operates as a supportive framework for self-help, with coaches guiding clients in formulating their own solutions to (professional) challenges. At its core, coaching therefore revolves around the facilitation of change for the client. However, attempting to encapsulate the multifaceted nature of change within the coaching context presents a formidable challenge. Change unfolds diversely across coaching scenarios and varies for each individual client, contingent upon specific contexts and circumstances. Consequently, defining change in coaching proves inherently elusive. To this day the concept remains largely uncharted territory. For this reason, the focus of the interdisciplinary research project “Questioning Sequences in Coaching” (QueSCo)^1^ is primarily on the aspect of the proclaimed change potential of questions and questioning sequences. In the specific context of this case study and for the purpose of this article, we define change as the act of arriving at a new or different decision through reflection upon one’s own statements, behaviors, or viewpoints. This can be articulated as “change between the earlier and later stance of the client.” As this work will demonstrate, this transformation can occur by initiating a shift in perspective by the coach.

“(a)dequate reflection on one’s experience is often seen as a steppingstone to change because reflection can allow the client to construe his or her life and social relationships in additional and alternative ways” (Muntigl and Zabala, 2008, p. 188).

As Muntigl and Zabala (2008) point out, self-reflection is often regarded as a catalyst for transformation. It is considered to be a critical examination of oneself and one’s own thoughts and actions (Greif, 2008). The fact that self-reflection is an essential impact factor of coaching has already been proven several times (Greif, 2008, 2018). Nevertheless, there is still substantial research needed in the field of coaching process research to address how exactly self-reflection is elicited in coaching conversations and how it unfolds on a local level.

Since coaches do not provide their clients with direct solutions but rather assist in developing their own pathways to solutions (Coaching-Magazin, 2024), similar to psychotherapy, the central task of coaches is to get clients not only to verbalize their experiences, but also to reflect on themselves and their experiences (*cf.* Greif, 2008; Muntigl and Zabala, 2008; Mack et al., 2016). Questioning practices play a central role in facilitating this process:

“Questions initiate hypothetical imaginative processes that have an immanent tendency to turn into self-reflection processes. […] they are also designed to enable new experiences because they involve a change of viewpoints and perceptual perspectives”^2^ (Köller, 2004, p. 662).

Questioning practices have particular transformational powers in helping professions like coaching. Not only are questions regarded as a fundamental instrument for controlling and structuring the conversation (Deplazes, 2016; Jautz et al., 2023), they also enable coaches to evoke self-reflection processes in clients and thus drive the coaching-immanent change project (Graf and Spranz-Fogasy, 2018b; Spranz-Fogasy et al., 2019). Schreyögg (2012) therefore names asking questions as the most important task of a coach, while Tracy and Robles (2009, p. 131) also describe questioning as “one of, if not the, central communicative practice of institutional encounters.” Coaches have a wide repertoire of questioning actions to stimulate self-reflection in clients and thus successfully advance the coaching change project (Bercelli et al., 2008; Muntigl and Zabala, 2008; Graf and Spranz-Fogasy, 2018a; Spranz-Fogasy et al., 2019). This transformational power of questioning practices in coaching has been asserted in the practice literature for many years, but there is little empirical research on the change potential of reflection-stimulating techniques used by coaches (Peräkylä, 2019; Graf et al., 2023b; Fleischhacker and Graf, 2024). This article aims to contribute to closing this research gap.

However, as Marciniak et al. (2016) point out in the context of linguistic and conversation analytic psychotherapy research, questions aren’t the only instruments for the elicitation of change. They name questions as one out of four basic therapeutic activities (that can also be applied to other helping conversations such as coaching): Questions, formulations, interpretations and extensions. In the following questioning sequences under investigation, formulations will too play an important role alongside the respective questions. Weiste and Peräkylä (2013) developed a classification of formulations comprising four specific function types: highlighting formulations, rephrasing formulations, relocating formulations and exaggerating formulations. In particular, this paper will further explore rephrasing formulations and relocating formulations, both in which “[…] the therapist transforms the client’s account and adds some elements that were not originally in the client’s turn” (Weiste and Peräkylä, 2013, p. 306). Through a rephrasing action, an aspect that the coach considers to be particularly relevant for the coaching is thus brought to the center. “Rephrasing is used to switch to the level of subjective experience at points where the client is more fact-oriented in their narrative” (see text footnote 2, respectively) (Mack et al., 2016, p. 53f.). Relocating formulations on the other hand are typically used for pattern identification and to link two real events, usually from the past and the present.

Mack et al. (2016) conducted a study on the subject of whether the function types of formulations developed by Weiste and Peräkylä (2013) can also be applied to questions. They came to the conclusion that the functions of formulations can also be observed in questions asked in psychotherapy. They also concluded that “[…] the connection between formulations and questions goes even further: beyond structural and functional similarities, the two often occur in combination” (see text footnote 2, respectively) (Mack et al., 2016, p. 91). This is precisely where the present work comes into play. The object of investigation in this paper are therefore questioning sequences from authentic coaching data in which a coach asks a question immediately after a rephrasing and/or (hypothetical) relocating action. The goal is to analyze how the questions further facilitate the hypothetical imaginative process through a change of perspective and how exactly they elicit self-reflection processes in the clients. This article delves into the intricate interplay between coaching, questioning practices, and the elusive concept of change through self-reflection, aiming to shed light on the nuanced linguistic dimensions that shape the coaching process.

## 2 Data and method

The subject of the study is a coaching process consisting of three sessions between a female coach and a female client. The durations of the sessions at hand are as follows: the first session has a total length of 1 h, 40 min, and 15 s; the second session is 1 h, 19 min, and 13 s long, and the third and final session lasts for 1 h, 20 min, and 32 s. The coach has a diploma in economics and an education as a systemic coach, working in the realm of solution-oriented, business-oriented systemic coaching. The client, a soft skills trainer at a university with a master’s degree, is unsatisfied with her job, seeking new challenges. The goal of the coaching is therefore for her to figure out where her professional journey is going and what her next steps should be. Recently she has been unsuccessful in job applications, impacting her self-confidence. She is also considering further training while job hunting.

The selection of this particular dyad is based on the deliberate choice to conduct a single case study. This case study aims to exemplify a specific type of questioning practice within coaching conversations. The intention is to reveal typical patterns and structures that can serve as paradigmatic observations in other coaching conversations, laying the groundwork for future research (*cf.* Lamnek, 1993, p. 16). This process was chosen due to the repetitive use of the specific questioning format by the coach, indicating its incorporation as a consistent element in the coach’s repertoire of actions. Furthermore, the client actively engages with this form of questioning, providing syntactically fitting and conditionally relevant responses. Thus, this process offers a particularly rich context for observing and analyzing the phenomenon in question. Each of the three questioning sequences is representative of a type of questioning that is applied multiple times throughout the process.

The chosen process was collected as part of the interdisciplinary research project “Questioning Sequences in Coaching” (QueSCo).^3^ The QueSCo research corpus consists of a total of 14 authentic coaching processes from different coaches and clients with a total of 50 sessions, where a process usually has between 3 and 4 sessions and one session lasts between 60 and 90 min. The corpus consists entirely of work-related coaching processes from Germany and Switzerland, that were video- and audio recorded and took place either face-to-face or online. The sessions were transcribed according to GAT2 and published as a cGAT minimal transcript (Selting et al., 2011; Schmidt et al., 2015), as GAT2 is the preferred transcription system in Germany. It is also machine-readable as a cGAT system and thus usable for quantitative evaluations, and for this reason was also used in the QueSCo project. The analysis was conducted on the original data. For the purpose of this paper the respective transcript excerpts have been translated into English. Original data is available upon request. Written informed consent for the publication of anonymized data was obtained from all participants. Names, organizations, places etc. referred to within the coaching have been replaced (see the QueSCo website for more information).

**Figure 1:**
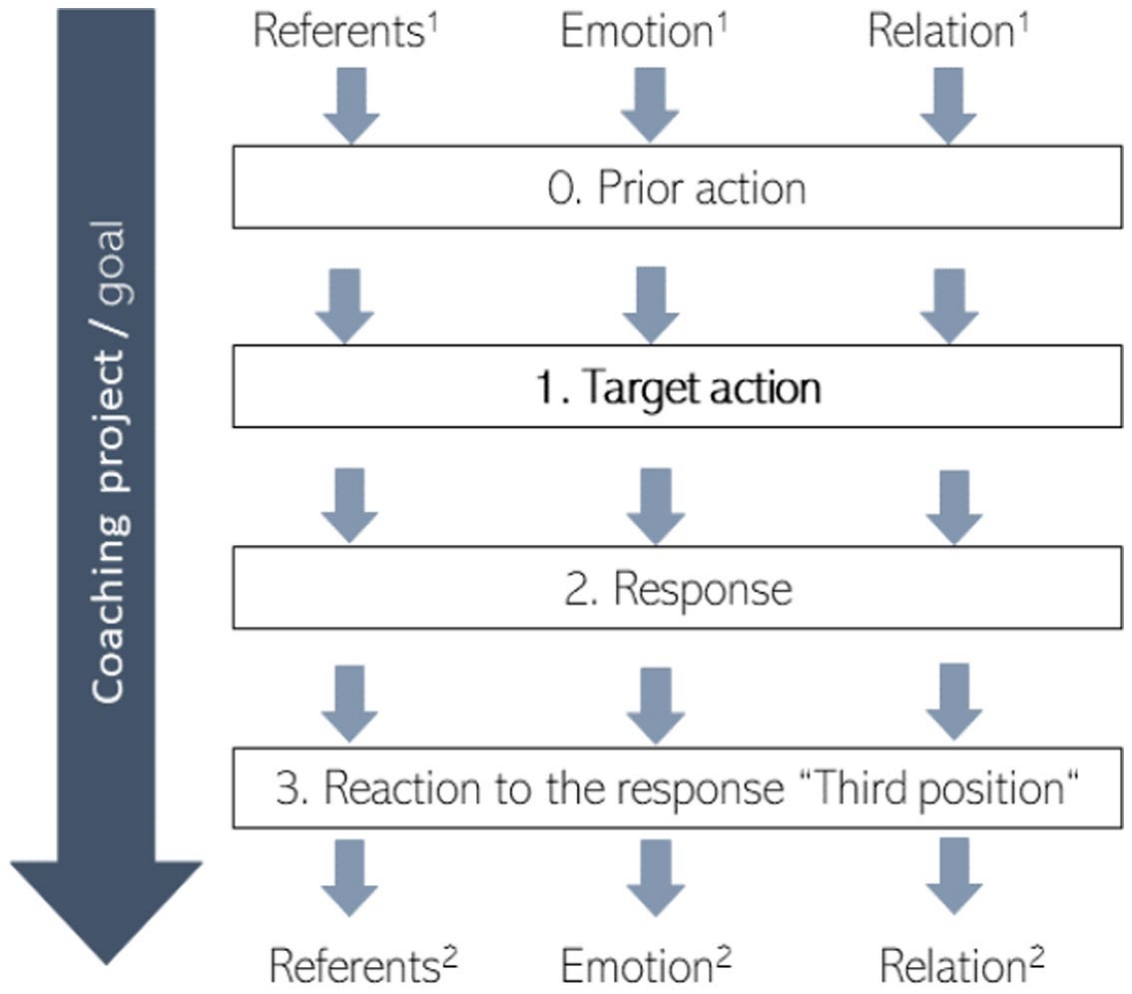
Transformative Sequences based on Peräkylä (2019, p. 267) and adapted to the coaching context.

Following Peräkylä’s Transformative Sequences model (see Figure 1), this paper will conduct a complete sequence analysis of three questioning sequences to investigate the transformative power of the respective sequences. Like Peräkylä (2019) we apply the unique method of Conversation analysis, as “[t]he central tenet of CA is that conversation is sequentially organized” (Stivers, 2013, p. 191). The focus lies on the first position (the coach’s utterance), the second position (the client’s response) and the third position (the coach’s reaction to the response). As Peräkylä (2019) also recognizes in his Transformative Sequences model, looking at the prior actions can also be of importance in sequence analysis, as they can provide information about the motivation and triggers of the coach’s questions. Therefore, in the typology of questioning sequences developed in the research project “Questioning Sequences in Coaching” (QueSCo)^4^, the two positions prior to the target action are always considered as well. In this paper, however, due to limited space, prior actions are only paraphrased at relevant places and are not included in the transcript excerpts, therefore following Schegloff’s (2007) understanding of a sequence consisting of three turns.

In the findings chapter, a full sequence analysis will be presented for all three cases. Case 1 is a questioning sequence from the first session, while cases 2 and 3 are extracted from the second session of the coaching. For a better step-by-step understanding of the sequence analysis, each of the cases will be subdivided into the analysis of the first, second and third position. The first position is the initiating turn in which the coach asks a question immediately after a rephrasing/relocating action. In the second position, the client’s answer will be examined with regard to recognizable elements of self-reflection. In the analysis of the third position we look at how the coach reacts to the client’s answers and whether she accepts them as appropriate. Finally, in the discussion part at the end of the paper, the findings of the analysis will be summarized and discussed.

## 3 Findings

The following analysis will examine a specific questioning practice that a coach used several times during the whole coaching process. In the selected cases, after a short rephrasing action at the beginning of the turn, where the coach briefly rephrases some of the client’s prior statements, the coach uses a hypothetical relocating action, prompting the client to “imagine” herself in a specific scenario. The hypothetical scenario introduced by the coach aims to initiate a change of perspective in the client. The coach finally ends her turn by asking the client a question, leading the client to verbally comment her thoughts on the scenario. The assumption is that the introduction of a hypothetical scenario, in combination with a subsequent question, imposes a constraint for the client to verbally asses and reflect on her own thoughts and statements. By looking at the client’s answers and determining whether (1) the client follows the constraint to critically assess the hypothetical scenario and therefore her own statements and (2) whether the client’s answers show elements of self-reflection, we will try to find an answer to the question whether the questioning practice at hand has a reflection-stimulating potential.

### 3.1 Case 1

Since completing her master’s degree, the client has been employed as a soft skills trainer at a university. Over time, she has become dissatisfied with her current position, sensing a lack of challenge and a professional standstill. Consequently, she is now contemplating the direction of her career journey and considering her next steps. Despite ongoing attempts to apply for alternative job opportunities, she has faced consistent setbacks, adversely affecting her self-confidence. Prior to the following sequence, the client explains how in her current job, she does not feel appreciated and seen for all the work that she does for her team. After the coach asks her if she has already experienced similar situations in her life, the client continues to explain how during her studies, she was always the one to do most of the work in group projects, which made her feel as unappreciated as she does now.^5^

**Table 1:** 

**2397**	**CO3**	so i can hear out of it that you have °h a high standard o of yourself and your work yes and um °hh and that is
**2398**		(0.3)
**2399**	**CO3**	in this study situation a little like here as well [it has] triggered similiar feelings ((smacks)) °hh um and and has um [contri]buted to this feeling of discomfort right so you no longer felt comforta[ble] °hh and now your studies are over and this work project as well and you are now in a position in a professional environment °hh where this is still present right
**2400**	**KL1**	[hmhm]
**2401**	**KL1**	[yes]
**2402**	**KL1**	[yes]
**2403**		(0.39)
**2404**	**CO3**	°h um (.) and now (.) this fantasy imagine you come home tonight and say to your boyfriend you
**2405**		(0.28)
**2406**	**CO3**	i quit my job today
**2407**		(0.21)
**2408**	**CO3**	[when you] hear that coming out of my °h [mouth] like that what
**2409**	**KL1**	[hmhm]
**2410**	**KL1**	[h°]
**2411**		(0.24)
**2412**	**CO3**	what is the very first thought that comes to your mind
**2413**		(1.98)
**2414**	**KL1**	the very first thought is really such a
**2415**		(0.31)
**2416**	**KL1**	ah there i get
**2417**		(0.29)
**2418**	**KL1**	for a moment h° uhm sh my breath stops because °hh i get like a
**2,419**		(1.26)
**2420**	**KL1**	(xxx) and i °hh would have a difficulty quitting without having a new job
**2421**		(0.71)
**2422**	**CO3**	ah that is
**2423**		(0.21)
**2424**	**CO3**	yes
**2425**		(0.24)
**2426**	**KL1**	yes (.) that (.) [i]
**2427**	**CO3**	[i think that_s] a very important [and (xxxxxxxxx reason) yes]
**2428**	**KL1**	[yea i think that would] not work
**2429**		(0.65)
**2430**	**KL1**	that could or like what does it mean that would not work of course it would work but I think
**2431**		(2.43)
**2432**	**KL1**	that on the one hand and on the other hand also i
**2433**		(0.38)
**2434**	**KL1**	yea no that_s actually it yea
**2435**		(0.35)
**2436**	**CO3**	hmhm
**2437**		(0.82)
**2438**	**KL1**	and also to justify it i think i (.) always feel like in front of other people i still have to justify myself in front of my colleagues in front of my friends [and s]o on and then to say °h what you quit your job and still do not have a new one i think that would be so hard for me too
**2439**	**CO3**	[yes]
**2440**		(0.26)
**2441**	**CO3**	yes
**2442**		(0.89)
**2443**		((ringing in the background))
**2444**	**CO3**	well i (.) now hear a very important sentence for a moment my breath stops [analogous]ly speaking yea °h and i would not quit without having a new job
**2445**	**KL1**	[hmhm]
**2446**	**KL1**	yes
**2447**		(2.85)
**2448**	**KL1**	yes
**2449**		(2.15)
**2450**	**CO3**	what does the statement mean to you

#### 3.1.1 First position (line 2397–2412)

In line 2397 the coach begins her turn with the introductory statement “so i can hear out of it that (…).” The coach indicates to her client that she is not simply reproducing what the client said, but rather how she understood the client’s statements, whereby she incorporates her own understanding and interpretation of the statement. In doing so, the coach uses terms that the client herself had used several times in the prior actions leading up to the first position, such as “feeling” and “standard.” In line 2,399, the coach explicitly relates the past to the present (“and you are now in a position in a professional environment °hh where this is still present“). The coach thus redirects the focus away from the past and back to the present. She then introduces a hypothetical scenario with “and now (.) this fantasy imagine”(line 2404). By saying “and now this fantasy,” an immediate transition to a “new” fact is introduced. The abrupt transition suggests that there is a connection between the current topic and the following scenario. “and now” thus serves as a connector between the rephrasing action and the hypothetical scenario that follows. At the same time, the conjunction and the adverb serve to “focus attention” (see text footnote 2, respectively) (Spranz-Fogasy, 1986), as the coach thus signals to the client that a transition to a new issue follows next. The explicit request to “imagine” encourages the client to think about the hypothetical scenario. Subsequently, in line 2,406, the coach uses direct speech (“i quit my job today”) and uses the first person singular, demonstrating closeness. The use of the first person singular form here potentially allows the client to put herself in the hypothetical scenario more easily. By introducing a new, hypothetical scenario and explicitly asking the client to imagine herself in this scenario, the coach creates a new approach to the topic. A change of perspective is encouraged – from the status quo to a new, different, hypothetical state.

Immediately after the request to the client to put herself in the hypothetical scenario described, the coach introduces the question with “[when you] hear that coming out of my °h [mouth]” and thus formulates the question as the second part of a conditional structure. Through the anaphoric reference of the sentence (“[when you] hear ***that*** coming out of my °h [mouth] like that”), the question is finally linked to the hypothetical scenario “i quit my job today”(line 2406). In this way, the coach explicitly refers to the scenario she described. This has a guiding function, because the coach indicates that there is a logical connection here. Finally, in line 2412, the open wh-question “what is the very first thought that comes to your mind” follows. The question about the “very first” thought signals to the client that she should express her thoughts directly and without delay, without thinking long and hard about the answer beforehand. She should answer intuitively or according to her gut feeling and “think out loud.” Although questions always have conditional relevance and impose a follow-up expectation on the answer, questions as part of a conditional structure (If (...) then) have an even stronger influence on the follow-up action, since they specify a certain framework within which the answer may move (*cf.* Klüber et al., 2012). In this case, the client is explicitly required to critically assess the hypothetical scenario. This means that the conditional structure not only has a guiding function, but also explicitly creates a constraint to make a critical assessment. The question can therefore be understood as a direct request or demand to verbalize the required (self-)reflection. The client recognizes this constraint and specifically aligns her response to this question.

#### 3.1.2 Second position: the client’s response (line 2414–2441)

Since a question always makes an answer conditionally relevant and self-reflection can only be examined by looking at the client’s reaction to the question, the next step is to look at the second position.

In case 1, the client gives her answer to the question “what is the very first thought that comes to your mind” after a pause of 1.98 s. She begins her turn with “the very first thought is” (line 2414). You can see that there is a direct reference to the question asked immediately before. The syntactically matching response indicates her understanding that she is supposed to provide an immediate answer and verbalize what she is thinking. After a short pause with a repair initiation, the client starts a new sentence. The statement “for a moment h° uhm sh my breath stops” also indicates that the answer is rather spontaneous and signals that this scenario described triggers strong (negative) emotions in her. At this point it becomes clear that the coach’s request for the client to explicitly verbalize her initial thoughts has been successful.

Several moments of self-reflection can also be identified in the client’s answer. For example, the client uses the epistemic sense and performative expression “i think” a total of four times (lines 2428, 2430, and 2438). This makes the subjectivity of her statements clear, as the client explicitly verbalizes that these are her own subjective opinions. The particles used can also be interpreted as signs of uncertainty which in turn refers to the spontaneity of the answer. All in all, the repeated use of “i think” indicates an initiated process of reflection. There are also several longer pauses during her turn (lines 2419, 2421, 2431, and 2437). In line 2,430 she also contradicts herself once (“or like what does it mean that would not work of course it would work but”), which again suggests a process of reflection on her own statements.

It is noticeable that the client makes self-initiated repairs at several points in her turn and interrupts her own train of thoughts several times. The fact that she does not find the right words at some points can be explained by the coach’s question and the explicit request to express the “very first thought.” In line 2,432, the client’s wording “that on the one hand” suggests that another reason or piece of information will follow. After the corresponding counterpart “on the other hand,” there is a short pause, whereupon the client takes back the statement and initiates a repair with “yea no that_s actually it yea.” Here you can see that the client is talking without knowing exactly what she wants to say or before she has sorted out her thoughts and found the appropriate words. After a pause and a positive feedback signal from the CO (“hmhm”), the second argument follows in line 2438, which the client now knows how to express. She continues her thoughts with the sentence “and also to justify it i think i (.) always feel like in front of other people.” The use of the adverb “always” is particularly interesting here. The client thus independently infers from the hypothetical scenario that has been discussed to several moments in her life when she feels as if she has to justify her decisions to other people. The hypothetical scenario introduced by the coach thus leads to an independent pattern identification on the client’s part. Overall, the client strongly orients and aligns her answer to the first position and fulfills the coach’s follow-up expectation.

#### 3.1.3 Third position: the coach’s reaction to the response

The coach’s reaction to the client’s response plays a pivotal role in recognizing the transformational power of questioning sequences, as it is the “place” where the coach either accepts a client’s response as appropriate and sufficient or flags it as inappropriate and insufficient (Sidnell, 2010; Graf et al., 2023a). If the coach accepts and ratifies the answer, they can move forward in the coaching process to further facilitate change, whereas if the coach decides that the client’s answer did not meet his or her expectations, the coach has various options to continue exploring the topic without moving forward in the conversation, e.g., by asking for a clarification or an elaboration, rephrasing the question or insisting on the question (see Graf et al., 2023a). Whether or not a questioning sequence can be defined as successful or unsuccessful therefore depends on the coach’s reaction to the client’s response.

After the client’s detailed answer to the question “[when you] hear that coming out of my [mouth] like that what °h what is the very first thought that comes to your mind” (lines 2408–2412), the coach picks up on the client’s statement that the thought of quitting made her breathless and that she “would have a difficulty quitting without having a new job” (lines 2418–2420), therefore starting the third position with a highlighting formulation (*cf.* Weiste and Peräkylä, 2013). She emphasizes and addresses the highlighting function of her statement on the meta-level with “i (.) now hear a very important sentence.” She then reproduces the client’s statement almost word for word. The coach ends her turn at this point without asking another question. The client therefore potentially does not know what is now being asked of her which can be seen in the long pauses and the two feedback signals “yes.” Only after a total pause of 5 s the coach continues talking and finally asks what the statement means to the client. Here again, there is a direct connection between the question and the highlighting formulation in line 2,444, which means that “the statement” again functions as a link between the formulation and the question. The question in line 2450 (“what does the statement mean to you”) finally forms the first position of a subsequent question sequence. In conclusion, the following can be said about the third position or about the entire questioning sequence: The client’s answer is accepted by the coach as an appropriate and sufficient fulfillment of her follow-up expectation. The next question, and thus the next question sequence, refers to a statement by the client that the coach considers important and therefore wants to go further into this aspect. The coach’s reaction to the client’s answer thus has a guiding and change-facilitating function and promotes the further course of the coaching conversation.

### 3.2 Case 2

A somewhat different approach to a change of perspective is facilitated by the coach in case 2. The client reports on a situation during a job application process not long ago. After a supposedly very good interview, she is assured by the company that they will get back to her with a decision in the course of the week. However, the company does not follow through with their promise which leads to dissatisfaction and frustration on the client’s part.

**Table 2:** 

**271**	**CO3**	i can well well empathise with that
**272**		(0.2)
**273**	**CO3**	°h and i_m just wondering if this story now um
**274**		(0.7)
**275**	**CO3**	well until that wednesday where lunch and then presentation [were yes] what you described at the beginning um °h
**276**	**KL1**	[hmhm]
**277**		(0.22)
**278**	**CO3**	that you are promised to receive a re[ply] that does not happen right and also the form uh of the reply is completely different and °hh (.) um imagine you want to tell this to a good friend and (.) no the other way around a good friend would tell you a story like this
**279**	**KL1**	[hmhm]
**280**	**KL1**	°hh
**281**	**CO3**	°h (.) try to think about it from the outside you are being told this story from the outside what would be your impression
**282**		(3.12)
**283**	**KL1**	well i f (.) the impression is super unprofessional i mean super unprofessional and (.) unappreciative
**284**		(0.26)
**285**	**KL1**	i mean
**286**		(1.12)
**287**	**KL1**	two (.) i mean hm i find that really in a large extend [((laughs))]
**288**	**CO3**	[yes]
**289**		(1.79)
**290**	**CO3**	and
**291**		(0.95)
**292**	**KL1**	yea my dad has said that before (.) when i applied somewhere else the (.) the
**293**		(0.31)
**294**	**KL1**	difficulty is that in such an application process it_s really no exception to be treated this way soun [soun sounds so]dramatic now but °h it is very often i think that one does not get any repl[y that people s]a[y]
**295**	**CO3**	[hmhm]
**296**	**CO3**	[hmhm]
**297**	**CO3**	[o]r that yea
**298**	**KL1**	yes exactly they will get in touch they then do not get in touch and °h (.) my dad once told me and i always try to tell that to myself like if one really wants to work in a company that
**299**		(0.33)
**300**	**KL1**	well (.) works like this because (.) i mean if they work like this in [their application process] they will probably also have a way of working like this normally and in this point i completely agree with him and °hh this was (.) well (.) i know that of course i still would have wished that everything would have gone differently [from the first thing that they] °h [well] would not have behaved so unprofessional but yea i [mean (.)]we are not at make a wish here anyway ((laughs, 1.23 s))
**301**	**CO3**	[((incomprehensible))]
**302**	**CO3**	[((laughs, 2.06 s))]
**303**	**CO3**	[yes]
**304**	**CO3**	[yes]
**305**	**CO3**	yes
**306**	**CO3**	well i can really understand you (.) that especially now since you were interested in the topics and you thought yes there is so much um that fits (.) you really wanted the job
**307**	**KL1**	yes
**308**	**CO3**	yes i can empathise with that really well °h (.) and at the same time there emerged such a such a feeling inside of me °h
**309**		(0.24)
**310**	**CO3**	hm
**311**		(0.29)
**312**	**CO3**	the so these whole premises tha [what you say] now as well that is what you considered unprofessional and not very ap[preciative] right °h that is also in the room and [i find] that quite good that you take notice of that and take it into a[ccount]
**313**	**KL1**	[hmhm]
**314**	**KL1**	[yes]
**315**	**KL1**	[yes]
**316**	**KL1**	[yes]
**317**		(0.93)

#### 3.2.1 First position (line 271–281)

The coach begins her turn in line 271 with an affiliative action (“i can well well empathise with that”), referring to the prior action in which the client explains the situation and expresses her frustration about it. The coach hereby shows the client that she supports her affective attitude (*cf.* Jefferson, 2002; Lindström and Sorjonen, 2012). In line 273, she introduces the next relocating action with “and i_m just wondering if this story now um” which is not continued after a pause of 0.7 s. Instead, she rephrases the client’s previous turn after a self-initiated repair. Subsequently, in line 278, the transition from the rephrasing action to a hypothetical scenario with the connector “and” follows, similar to case 1. Just like in the previous case, the immediate transition to a “new” fact suggests a consequential relation. As in case 1, “imagine” can be understood as an explicit request by the coach to think about the hypothetical scenario. Finally, a description of the announced scenario follows. The coach makes a mistake when describing the scenario, which is repaired by the explicit repair initiator “no the other way around”. Here it becomes clear that the perspective to be adopted is crucial for the scenario or for the question that follows in line 281.

A change of perspective is initiated in lines 278–281, by relocating the client’s frustration with the problem into a new, hypothetical scenario. By asking the client to take the perspective of a friend who is hearing this story for the first time, the coach tries to give the client a different approach to the story, as she should look at it “from the outside.” In line 281, she voices another explicit request to change the perspective (“try to think about it from the outside”). In case 2, as well as in case 1, the relocating action has a different quality than relocating according to Weiste and Peräkylä (2013), since the aim here is not to link two events that have actually taken place, but rather to relocate a currently discussed issue (i.e., a real point of reference) of the client to a hypothetical scenario. For the purpose of this paper we will therefore refer to this as “hypothetical relocating.” While relocating according to Weiste and Peräkylä (2013) is typically used for pattern identification, hypothetical relocating here has the specific function of a change of perspective. Both forms pursue the goal of stimulating (self-) reflection.

Albeit the structure of the question in case 2 deviates somewhat from the question in case 1, it is still similar in the way that an explicit request for a change of perspective (“try to think about it from the outside you are being told this story from the outside”) is instantly followed by the question “what would be your impression.” The structure of the question is similar to a conditional structure (according to the pattern: When you hear this story told from the outside, what would be your impression?). Although the change of perspective asks the client to take an outside view of her own story, the question “what would be your impression” still asks for her subjective assessment of the story. Here too, the combination of relocating action and question is an explicit invitation to verbalize one’s own thoughts and thus to (self-)reflect.

#### 3.2.2 Second position: the client’s response (lines 283–304)

After a pause of 3.12 s, the client begins her answer with “well i f” and does not pronounce the words “I find” or “I think.” Instead, she initiates a repair and restructures her sentence. She repeats the word “impression,” which the Coach uses in her question, and thus provides a syntactically appropriate answer in which the orientation toward the question is clearly visible. She highlights the word “super” in the statement “the impression is super unprofessional” and repeats the statement again immediately afterwards, adding “and (.) unappreciative.” After another repair, she again emphasizes her negative assessment with “i mean hm i find that really in a large extend,” which makes the client’s indignation about the company’s behavior even clearer. With her short and quiet laugh, she plays down the unpleasant topic. Between the lines 282 and 287 there are two repairs and several long pauses during the client’s turn. This indicates that the client is thinking about what she wants to say or how she should formulate her next thoughts. She uses the hesitation-indicating expressions “i mean” (line 285) and “and” (line 290) which are hesitantly intoned here, as gap fillers. This is followed by a longer pause before she goes ahead with her turn. It is recognizable that the client is addressing the coach’s question and thus the hypothetical scenario and is reflecting on her impression of the company’s behavior while she speaks.

However, the client does not elaborate on the change of perspective introduced by the coach in the first position. Instead of adopting the perspective of a friend who is being told this story for the first time by another friend, the client herself carries out a relocating activity by referring to an similar experience in the past in which her father asked her the question “if one really wants to work in a company that (…),” since the company will probably also have a similar way of working in other aspects. In this way, the client allows the perspective of another person to flow in, but not the perspective of a hypothetical friend, as the coach introduces in the first position, but rather the perspective of her father. The client finally comes to the conclusion “and in this point i completely agree with him.” The client’s single-handed linking of the hypothetical scenario with an event that took place in the past, as well as the implicit realization that the company’s behavior was unacceptable, can be seen as verbalized self-reflection.

#### 3.2.3 Third position: the coach’s reaction to the response (lines 305–316)

In reaction to the client’s response, the coach first expresses understanding and sympathy through affirmation (“well i can really understand you” (line 306) and “yes i can empathise with that really well” (line 308)). She again uses rephrasing formulations which can be clearly seen in the statements “and you thought” (line 306) and “tha [what you say] now as well” (line 312). In addition, the coach also praises the client in line 312: “[i find] that quite good that you take notice of that and take it into a[ccount].” The coach refers directly to the client’s previous turn, in which she responds to the question “what would be your impression” (line 281) by describing how much she considers the company’s behavior “unprofessional and (.) unappreciative” (line 283). The coach thus refers to the degree of self-reflection in the client’s answer and evaluates it positively. It can therefore be said that the client fulfilled the coach’s expectation. Overall, the sequence can be considered a successful questioning sequence in which the systematic use of a particular succession of relocating action and question achieves an answer in which the client shows a degree of self-reflection that is not only accepted by the coach in the third position, but also evaluated positively.

### 3.3 Case 3

In the next few turns, the coach and the client give further input on the hypothetical scenario and the overall matter. At some point the client says that she thinks that maybe she is just too ambitious and maybe she should be less demanding. The coach picks up on this statement and asks the client how she could have been less demanding, what would have changed as a result and why being less demanding and ambitious would have been a good way for her. After the client’s ambivalent answers, which are characterized by uncertainty, the coach again introduces a hypothetical future scenario, similar to the one in case 1.

**Table 3:** 

**376**	**CO3**	°h (.) and let_s pick pick up the thread so the this fantasy let_s assume you uhm (.) get the acceptance [that is a]great success [right you a]re rea[lly hap]py °h and then the next step goes
**377**	**KL1**	[hmhm]
**378**	**KL1**	[hmhm]
**379**	**KL1**	[hmhm]
**380**		(1.22)
**381**	**CO3**	and (.) um now imagine what you have already developed as a sense as a feeling for this company (.) um because of the way °h the employees there presented themselves to you °hh (.) um (.) and you go in into the work and notice these things there even more often (.) [i mean] there is a probability [given right]
**382**	**KL1**	[hmhm]
**383**	**KL1**	[yes (.) sure (.) of] course
**384**		(0.22)
**385**	**CO3**	and now once again (.) uhm (.) uhm
**386**		(0.46)
**387**	**CO3**	this this idea of yours (.) right that is maybe I have to just try it and not be so demanding how d how how how does it sound when I say that
**388**		(2.26)
**389**	**KL1**	well especially when you say that when you are in the working life and it will happen even more often it does not sound good at all and i
**390**		(1.54)
**391**	**KL1**	hm
**392**		(0.2)
**393**	**KL1**	°h i must say it always makes me think back to an experience i once had it was just a (working student position) well i was (.) I told you that for a longer time I was sick
**394**		(0.47)
**395**	**KL1**	and after that i
**396**		(0.26)
**397**	**KL1**	or like then after half a year i um applied for a (working student position)
		**(lines 398–405 omitted)**
**406**	**KL1**	i had an job interview there as well and it was really awful in the sense of (.)°h i just had the feeling that something wasn’t right like I couldn_t really say why but i just didn_t have a good feeling like °h (.) the tasks somehow matched and °h (.) like i said the whole values of the company also matched well and °h
		**(lines 407–422 omitted)**
**423**	**KL1**	[yes exactly some]how um (.) yes exactly and then at that time i thought um i did not have many alternatives i just wanted to do something because i was also °h a little bit
**424**		(0.26)
**425**	**KL1**	aimless so i thought oh i just do do it now because in the end it was a working student job the money didn_t matter i just wanted to try it °h (.) i did it then i quit again after a month
**426**		(0.71)
**427**	**KL1**	because i
**428**		(0.4)
**429**	**KL1**	said it is not for me (.) and it does not make any sense (.) and i do not feel comfortable i do not feel integrated into the team °h all these things and that after 6 weeks or so after a short period of time and i have never really done that after such a short period of time °hh and now i think about it from time to time (.) when i like you also said um (.) put myself in the situation that if i would be working there and it would be terrible °h then i think to myself (.) yes well but (.) theoretically my gut (.) feeling was always something i could
**430**		(0.45)
**431**	**KL1**	trust
		**(lines 432–450 omitted)**
**451**		(1.04)
**452**	**CO3**	yes we are now in this topic with the (.) with this current situation you have had the job interview after the last coaching and (if) the appointments in between now here (we) just plunged into this coaching session [very quickly] °h right and i would now like to go [back] a little °h (.) um
**453**	**KL1**	[((laughs))]
**454**	**KL1**	[yes]
**455**		(0.49)
**456**	**CO3**	and um (.) and reflect again (.) with you together (.) um (.) so in the
**457**		(0.2)
**458**	**CO3**	hm follow-up to the last session °h (.) in order to orientate yourself professionally and to find a direction for yourself °h what goal you [wou]ld like to set for the session today how would you like to use the session
**459**	**KL1**	[hmhm]
**460**		(0.2)
**461**	**KL1**	hmhm

#### 3.3.1 First position (lines 376–387)

By saying “and let_s (…) pick up the thread,” the coach announces that the topic will be further explored in the following. She introduces a hypothetical future scenario by saying “so the this fantasy let_s assume you uhm (.) get the acceptance,” which can be recognized by the terms “fantasy” and “acceptance.” Meanwhile, the client utters several affirmative feedback particles which signal that she agrees to devoting to the hypothetical scenario. In line 381, similar to the cases 1 and 2, the direct request (“and (.) um now imagine”) is followed by a detailed description of the hypothetical scenario in which the client more often notices the things she already perceived negatively on the behalf of the potential future employer. In lines 385–387, the coach finally initiates the relocating of the client’s statement by saying “and now once again […] this this idea of yours.” The coach then continues to reproduce the client’s prior statement that maybe she is just being too ambitious and maybe she should be less demanding, using the direct speech. She thereby takes the client’s statement, decontextualizes it and puts it in a new, hypothetical and future-oriented context in order to change the client’s perception of her own statement.

After the focus shift on the relocating action (“and now once again”) and the relocating action itself (“this idea of yours (.) right that is maybe I have to just try it and not be so demanding”), the coach finally follows up with the wh-question “how does it sound when I say that” (line 387). Again, the question has the form of a conditional structure, although posed with the premise placed last. The second part of the question “when I say that” shows an analogy to the formulation “[when you] hear that coming out of my °h [mouth]” from case 1. The request for an explicit change of perspective becomes clear at this point. As in case 1, the anaphoric reference (“how does it sound when I say ***that***”) makes it clear that the client should verbally state her opinion on the relocating action and that an explicit statement is required.

#### 3.3.2 Second position: the client’s reaction (lines 389–431)

After a pause of 2.26 s, the client gives a precise answer to the question: “well especially when you say that when you are in the working life and it will happen even more often it does not sound good at all.” Here, too, by saying “especially when you say that,” the client indicates that she is syntactically orienting her answer to the follow-up expectation of the question and that she understands what the coach is expecting or that she has interpreted the follow-up expectation correctly. It also becomes clear that the coach’s relocating action elicits a result-oriented reflection in the client, as the client comes to the conclusion that this hypothetical scenario does not sound good coming from the coach and that she does not agree with her own statement that she simply has to try not to be so demanding. She thus rethinks or reflects on her one statement and reassesses it, which ultimately leads to a change in stance. The reflection-stimulating potential of the systematic use of relocating action and the related question “how does it sound when I say that” becomes particularly clear in the client’s answer.

Similar to cases 1 and 2, the client fulfills the coach’s follow-up expectation and gives a precise answer to the question. With the statement “i must say it always makes me think back to an experience i once had,” the client additionally introduces an independent relocating. She states that this makes her think of an experience from when she was still a student, where she had taken a student job that she did not have a good feeling about from the start. While telling the story, she makes statements such as “i just had the feeling that something wasn’t right” and “i just didn_t have a good feeling” (406). The client continues to describe the situation from her past for about a minute (lines omitted) and finally makes the connection to her current professional situation in line 429: “and now i think about it from time to time (.) when i like you also said um (.) put myself in the situation that if i would be working there and it would be terrible °h then i think to mysel*f* (.) yes well but (.) theoretically my gut (.) feeling was always something i could (0.45) trust.”

The client directs the conversation from the hypothetical scenario established by the coach to a similar experience from her own past, and finally back to her current situation. In doing so, she implicitly comes to the conclusion that she should trust her gut feeling, as she did back then, and therefore should not try to be less demanding or to lower her expectations of a job. The initial relocating and accompanying pattern identification by the client herself are very central characteristics of successful self-reflection here.

#### 3.3.3 Third position: the coach’s reaction to the response (lines 452–461)

Surprisingly, the coach does not react to the client’s answer at all and instead carries out an agenda-thematizing action without further addressing the client’s response. The motivation for this intervention is not traceable in the conversation and can be explained by the epistemic authority of the coach in the coaching process (Dionne, 2021). The non-judgment of the client’s answer and the initiation of a new, higher level activity can be interpreted as “ratification qua accomplishment” (see text footnote 2, respectively) (Spranz-Fogasy, 1986), since it can be assumed that the coach judges the client’s contribution as an adequate answer to her question that does not require explicit ratification. It can therefore be assumed that the question sequence was considered successful by the coach, so that she can move the conversation and thus the coaching project forward.

## 4 Discussion

On the basis of three different questioning sequences, this paper examined a specific questioning practice that a coach used several times during a coaching process. The aim was to find out whether the questioning practice has a reflection-stimulating potential. In the selected examples, after a short rephrasing action at the beginning of the turn, the coach uses a hypothetical relocating action. The transition from rephrasing to the hypothetical scenario happens immediately and is facilitated by a connector (e.g., “and now”) which suggests to the client that there is a subsequent connection. At the same time, the conjunction and the adverb serve to focus the attention to what comes next. By the use of terms such as “fantasy,” “assume” or “imagine,” the coach also signals that a hypothetical scenario is being introduced. Supporting this, the coach uses direct and explicit prompts, such as “now imagine,” so that the client has no choice but to imagine herself in the scenario. In cases 1 and 3, the coach also creates a distance between the client and her statements by using the first person singular several times in the hypothetical relocating actions. This helps the client hear her own statement coming from another person, theoretically making it easier for her to look at her own statement from an outside point of view. In all of the three cases, the hypothetical scenario introduced by the coach aims to initiate a change of perspective in the client, paving the way for the question that finally leads the client to explicitly comment on the scenario.

In all of the three cases, the questions are posed as conditional structures, which all reveal syntactic and systematic similarities. The questioning pattern (When (...) then?) has a strong guiding function and places a strong consequential expectation on the client’s answer which was referred to in this paper as a constraint for critical assessment. In the examples, the question always makes an anaphoric reference to the hypothetical relocating action (e.g., “[when you] hear ***that*** coming out of my °h [mouth],” case 1, line 2408) which illustrates the systematic relationship between the hypothetical scenario and the question. The client is thus shown that there is a logical connection here. A change of perspective, and therefore a change in stance, was achieved by the coach explicitly asking the client to speak her thoughts aloud when she hears her own story or statement coming from the coach’s mouth. The question can therefore be seen as a request to verbalize the reflection process. The question about the “very first” thought also signals to the client that she should express her thoughts directly and without delay, without thinking long and hard about the answer beforehand. The client subsequently answers intuitively or according to her gut feeling.

When looking at the second position, it became apparent that the client recognizes the constraint for critical assessment that has arisen and orients her answers to it by providing syntactically matching answers and also picking up the wording of the question. In case 3, for example, the client answers to the question “how does it sound when I say that” (line 387) with “well especially when you say (...) it does not sound good at all” (line 389). It is clearly recognizable that the change of perspective, which is aspired by the question, is successful and thus a self-reflection process is elicited. In the client’s answers, other phenomena of self-reflection could also be observed, such as the frequent use of epistemic sense and performative expressions like “actually,” “maybe,” as well as “I find” and “I think.” Frequent repair initiators, long (thinking) pauses and the use of delay signals are also signs of a reflection process taking place. Another sign of self-reflection is the fact that the client contradicted her own statements soon after stating them aloud, therefore critically assessing them.

Another crucial aspect of self-reflection involves the independent pattern identification which can particularly be found in cases 2 and 3. It can be observed that in her answer to the question “what would be your impression” (case 2) the client independently uses a relocating action and establishes the link from the hypothetical scenario to an event from her own past in which her father gave her advice that can also be transferred to the current situation. This is very similar to case 3, where in her answer to the question “how does it sound when I say that,” the client again refers to an event in her past and comes to the own conclusion: “theoretically my gut (.) feeling was always something i could (0.45) trust.” The independent pattern identification initiated here by a relocating action and the coach’s questions is a crucial aspect of self-reflection and a convincing argument for the reflection-stimulating potential of the systematic use of hypothetical relocating and questioning.

The extent to which the coach assesses the client’s response as appropriate and whether the change project is moved forwards or stopped was examined in the third position. In all three cases it becomes clear that the coach evaluates the client’s answer as an appropriate fulfillment of the follow-up expectation of the question and that the change project is thus advanced. This is shown by the fact that in case 1, the coach navigates the conversation by highlighting an aspect of the client’s answer. In case 3, a new higher-level activity, an agenda-thematizing action, is initiated and in case 2, the client’s answer is even followed by a verbal, positive evaluation of the client’s answer and the degree of her self-reflection.

## 5 Conclusion

As shown in this article, hypothetical relocating can encourage reflection on the client’s own narrative and their own choice of words. In combination with a question, the coach’s action is finally transformed into a request for the client to explicitly verbalize and thus to critically assess their own thoughts. The systematic use of formulation and questioning thus has a reflection-stimulating potential and is therefore a significant tool for eliciting self-reflection, which is identified as a pivotal factor in advancing the overarching goal of coaching – facilitating change in clients. The paper calls for further exploration of the change potential immanent to coaching, emphasizing the need for continued research on the transformative power of questioning practices. In essence, the study illuminates the intricate dynamics of coaching, showcasing how coaches can shape self-reflection and contribute to the facilitation of transformative change in the coaching process.

## Data availability statement

The original contributions presented in the study are included in the article/supplementary material, further inquiries can be directed to the corresponding author.

## Ethics statement

Ethical approval was not required for the study on human participants in accordance with the local legislation and institutional requirements. The participants provided their written informed consent to participate in this study.

## Author contributions

CM and TS-F contributed to the article and approved the submitted version and examined the corpus for the occurrences of questions and formulations together. CM was mainly responsible for the detailed analysis of the chosen examples as well as the writing of the introduction and the discussion and conclusion. TS-F substantially contributed to the article by guiding, consulting and critically discussing the findings and writing with CM. All authors contributed to the article and approved the submitted version.

## Footnotes

^1^ https://questions-in-coaching.aau.at/

^2^Translated by authors.

^3^ https://questions-in-coaching.aau.at/results/

^4^ https://questions-in-coaching.aau.at/results/

^5^In the following excerpts, simultaneous utterances of the interlocutors are marked with square brackets. In the case of longer utterances by one speaker, the respective utterances of the other speaker are listed in the actual sequence in separate lines below the field of the first speaker.
